# IFNγ directly counteracts imatinib-induced apoptosis of primary human CD34+ CML stem/progenitor cells potentially through the upregulation of multiple key survival factors

**DOI:** 10.1080/2162402X.2022.2109861

**Published:** 2022-08-12

**Authors:** Dorina Ujvari, Alena Malyukova, Ana Zovko, Elham Yektaei-Karin, Harsha S Madapura, Marton Keszei, Noemi Nagy, Kourosh Lotfi, Niclas Björn, Jonas Wallvik, Leif Stenke, Daniel Salamon

**Affiliations:** a Department of Women’s and Children’s Health, Karolinska Institutet, Stockholm, Sweden; b National Pandemic Center, Department of Microbiology, Tumor and Cell Biology, Karolinska Institutet, Stockholm, Sweden; c Department of Cell and Molecular Biology, Karolinska Institute, Stockholm, Sweden; d Division of Hematology, Karolinska University Hospital Solna, Stockholm, Sweden; e Department of Medicine Solna, Karolinska Institutet, Stockholm, Sweden; f Department of Microbiology, Tumor and Cell Biology, Karolinska Institute, Stockholm, Sweden; g Department of Hematology, Linköping University Hospital, Linköping, Sweden; h Department of Medical and Health Sciences, Linköping University, Linköping, Sweden; i Department of Biomedical and Clinical Sciences, Linköping University, Linköping, Sweden; j Department of Public Health and Clinical Medicine, Umeå University, Umeå, Sweden

**Keywords:** Chronic myeloid leukemia, interferon gamma, apoptosis, leukemic stem cell

## Abstract

Tyrosine kinase inhibitors (TKIs) have dramatically improved the survival in chronic myeloid leukemia (CML), but residual disease typically persists even after prolonged treatment. Several lines of evidence suggest that TKIs administered to CML patients upregulate interferon γ (IFNγ) production, which may counteract the anti-tumorigenic effects of the therapy. We now show that activated T cell-conditioned medium (TCM) enhanced proliferation and counteracted imatinib-induced apoptosis of CML cells, and addition of a neutralizing anti-IFNγ antibody at least partially inhibited the anti-apoptotic effect. Likewise, recombinant IFNγ also reduced imatinib-induced apoptosis of CML cells. This anti-apoptotic effect of IFNγ was independent of alternative IFNγ signaling pathways, but could be notably diminished by STAT1-knockdown. Furthermore, IFNγ upregulated the expression of several anti-apoptotic proteins, including MCL1, PARP9, and PARP14, both in untreated and imatinib-treated primary human CD34+ CML stem/progenitor cells. Our results suggest that activated T cells in imatinib-treated CML patients can directly rescue CML cells from imatinib-induced apoptosis at least partially through the secretion of IFNγ, which exerts a rapid, STAT1-dependent anti-apoptotic effect potentially through the simultaneous upregulation of several key hematopoietic survival factors. These mechanisms may have a major clinical impact, when targeting residual leukemic stem/progenitor cells in CML.

## Introduction

Chronic myeloid leukemia (CML) is characterized by the reciprocal translocation t(9;22), which forms the Philadelphia chromosome. As a result, the fusion oncogene, BCR-ABL1 is formed and translated into a constitutively active tyrosine kinase, which plays an essential role in the pathogenesis of the disease.^1,2^ Inhibition of BCR-ABL1 activity by long-term administration of imatinib or other specific tyrosine kinase inhibitors (TKIs) has revolutionized the treatment of CML. A previously almost universally fatal malignancy has now been converted into a chronic disorder with near normal life expectancy.^3,4^ However, even after more than decade-long periods of continuous TKI administration, the majority of CML patients cannot stop their treatment without disease relapse.^5^ One explanation, perhaps the most plausible, to this limited potential of achieving cure with only TKI, is resistance of the CML stem/progenitor cell population to ABL1-specific TKI monotherapy. Such resistance can be mediated by either a BCR-ABL1-dependent mechanism or by the intrinsic or microenvironment-induced activation of various signaling pathways, which are also required for the survival of normal hematopoietic cells.^1,2^

TKI treatment can affect a broad range of immune cells, including dendritic, T and natural killer cells. Although TKIs mainly inhibit the function of these cells *in vitro*, due to the complexity of the immune system, TKI treatment *in vivo* upregulates interferon γ (IFNγ) production, especially in the tumor cell microenvironment.^6^ Accordingly, serum IFNγ levels have been shown to significantly increase during TKI treatment of CML patients in chronic phase.^7,8^

Although, in most cancer types, IFNγ exerts strong anti-tumorigenic effects,^9,10^ several lines of evidence suggest that the increased production of IFNγ during TKI treatment might play a negative effect on the therapeutic response of CML patients. For example, in CML patients the ratio of IFNγ positive T cells significantly increased during imatinib treatment, and was continuously elevated in patients without a major cytogenetic response, while in major responders the proportion returned toward values obtained in healthy controls.^11^ Furthermore, Held *et al*.^12^ showed in transwell experiments that soluble factors secreted by phorbol myristate acetate/ionomycin-activated T cells or interleukin (IL)-12/IL18-activated NK cells significantly inhibited the TKI-induced apoptosis of CML cell lines and peripheral blood mononuclear cells (PBMCs) of CML patients in chronic phase. Since only IFNγ (one of the most abundantly secreted soluble factors of activated T and NK cells), but not IFNα, tumor necrosis factor α, IL6, IL12, granulocyte-macrophage colony-stimulating factor (GM-CSF) or soluble CD40 ligand exerted an anti-apoptotic effect on TKI-treated CML cells, the authors suggested IFNγ as the key anti-apoptotic factor of the activated T and NK cell secretome.

We have previously shown that IFNγ upregulated several anti-apoptotic members of the BCL2 and BIRC (baculoviral IAP repeat containing) gene families, including the long isoform of MCL1 (MCL-1L) in the imatinib-treated CML cell line JURL-MK1. Accordingly, the anti-apoptotic effect of IFNγ on JURL-MK1 cells was counteracted by the presence of the selective MCL1 inhibitor A-1210477, suggesting that IFNγ might exert its anti-apoptotic effect on CML cells through the upregulation of MCL-1L.^13^

Based on these observations, our primary aims were to directly assess whether the inhibition of the pro-apoptotic effect of imatinib by activated T cell secretome depends on IFNγ, and to characterize the effect of IFNγ on the imatinib-induced apoptosis of CML cells, including primary human CD34+ CML stem/progenitor cells.

## Materials and methods

### Cell culture and reagents

The JURL-MK1^14^ and K562^15^ CML lines were obtained from the German Collection of Microorganisms and Cell Cultures (DSMZ) and cultured in RPMI 1640 medium containing 10% heat-inactivated fetal bovine serum, and 1 mM L-glutamine (complete medium). The cell lines were PCR-tested and found to be mycoplasma free.

Recombinant human IFNγ (PeproTech) was dissolved as recommended by the manufacturer. Imatinib (17 mM stock; Sigma-Aldrich) and ralimetinib (10 mM stock; Selleckchem) were dissolved in water. Wortmannin (10 mM stock; Selleckchem), SCH772984 (10 mM stock; Selleckchem), JNK-IN-8 (25 mM stock; Selleckchem), and SC75741 (20 mM stock; Selleckchem) were dissolved in dimethyl sulfoxide (DMSO). DMSO concentration was equalized in each well of a particular experiment.

### Isolation of primary human CD34+ CML stem/progenitor cells

PBMCs were separated from peripheral blood of untreated CML patients at the time of diagnosis (chronic phase of the disease) by leukapheresis. CD34+ cells were purified from PBMCs using the Dead Cell Removal Kit (Miltenyi Biotec) and the human CD34 MicroBead Kit (Miltenyi Biotec). Flow cytometric analysis using FITC-conjugated mouse anti-human-CD34 (clone AC136; Miltenyi Biotec; Cat. No.: 130–081-001), or FITC-conjugated mouse IgG2a (isotype control; Miltenyi Biotec; Cat. No.: 130–091-837) antibodies confirmed that in all cases more than 95% of the isolated cells were CD34+.

All human CML samples were obtained with informed consent and were used in accordance with the Declaration of Helsinki. Regional Ethical Committees at Stockholm and Linköping approved the study (registration Nos.: 99–146, 2016/42-31/4, and 2017/384-31).

### Production and purification of T cell-conditioned medium

PBMCs were separated from buffy coats of healthy blood donors by Ficoll-Paque density gradient centrifugation. T cells from PBMCs were enriched by negative selection using CD19 Pan B Dynabeads (ThermoFisher Scientific; Cat. No.: 11143D) according to the manufacturer`s instructions, followed by the removal of cells adherent to tissue culture dishes. T cells were seeded at 10^6^ cells/ml density in complete or StemSpan SFEM medium and were left untreated or activated for 48 hours by Human T-Activator CD3/CD28 Dynabeads (ThermoFisher Scientific; Cat. No.: 11161D; 1 bead/1 T cell). Conditioned medium was purified by pelleting the cells with centrifugation, followed by magnetic cleanup of beads and filtration of the supernatants through a 0.22 µm pore size filter.

### Culture of CML cells with isotype control or neutralizing antibodies in the presence of T cell-conditioned medium with or without imatinib

JURL-MK1 cells seeded at 8 × 10^4^ (batch No. 1) or 3 × 10^4^ (batch No. 2) cells/well or CD34+ CML stem/progenitor cells seeded at 3 × 10^4^ cells/well in 96-well plates were cultured for the indicated hours in the indicated ratios of a mixture of fresh complete or StemSpan SFEM medium and non-activated or activated T cell-conditioned medium (TCM), in the absence or presence of the indicated concentrations of imatinib and isotype control (mouse IgG1, κ (clone MOPC-21) and/or rat IgG2a, κ (clone RTK2758); both from BioLegend) and/or neutralizing mouse anti-human IFNγ (clone B27; BioLegend) and/or neutralizing rat anti-human GM-CSF (clone BVD2-21C11; BioLegend) antibodies. TCM was incubated with the antibodies for 30 minutes at room temperature, before adding to the cells.

The proportion of active caspase-3 positive (apoptotic) cells was quantified by flow cytometry. The ratio of active caspase-3/7 positive (apoptotic) cells was quantified by fluorescence live cell microscopy, in which the culture medium was supplemented with 2.5 μM IncuCyte Caspase-3/7 Green Reagent for Apoptosis (Essen Bioscience). The number of viable cells was manually counted using a hemocytometer and trypan blue exclusion, performed blind by two independent observers.

### Transient transfection of siRNAs

Negative Control No. 1, or STAT1 (s277) specific Silencer Select siRNAs (Cat. Nos.: 4390843 and 4390824; Invitrogen) were transiently transfected into JURL-MK1 cells with the Nucleofector system (Amaxa) using solution V with program T-16 according to the manufacturer’s instructions. Forty hours after transfection, dead cells were eliminated by the Dead Cell Removal Kit, and then the cells were seeded and treated as described. STAT1 knockdown efficiency was analyzed by immunoblot using cell lysates prepared immediately before the indicated treatment.

### Treatment of CML cells with small molecule inhibitors and/or IFNγ

JURL-MK1 cells transfected with control- or STAT1-specific siRNAs were seeded in complete medium, at 8 × 10^4^ cells/well in 96-well plates and then left untreated or treated for 18 hours with 1 μM imatinib and/or 5 ng/ml IFNγ, followed by the quantification of the proportion of apoptotic cells by flow cytometry.

JURL-MK1 cells seeded in complete medium, at 8 × 10^4^ cells/well in 96-well plates were pre-incubated with the indicated concentrations of ralimetinib, wortmannin, SCH772984, JNK-IN-8, SC75741 or solvent (in the absence or presence of 1 μM imatinib) for 1 hour, and then left untreated or treated with 5 ng/ml IFNγ for 18 hours, followed by the quantification of the proportion of apoptotic cells by flow cytometry or fluorescence live cell microscopy.

JURL-MK1 cells seeded at 3 × 10^4^ cells/well or K562 cells seeded at 2 × 10^4^ cells/well in 96-well plates, in phenol-red free complete medium supplemented with 1.25 μM IncuCyte Caspase-3/7 Green Reagent for Apoptosis (Essen Bioscience), were left untreated or treated with 1 μM imatinib and/or 5 ng/ml IFNγ, followed by the quantification of the proportion of apoptotic cells by fluorescence live cell microscopy.

Primary human CD34+ CML stem/progenitor cells seeded in StemSpan SFEM medium (StemCell Technologies), at 6 × 10^4^ cells/well, in 96-well plates were pre-incubated with 10 μM A1210477 or solvent (in the absence or presence of 5 μM imatinib) for 1 hour, and then left untreated or treated with 5 ng/ml IFNγ for 24 hours, followed by the quantification of the proportion of apoptotic cells by flow cytometry.

Primary human CD34+ CML stem/progenitor cells seeded in StemSpan SFEM medium without supplementation or supplemented with early acting cytokines^16^ (recombinant human Flt3-ligand (FL), stem cell factor (SCF) and thrombopoietin (TPO); 100 ng/ml each; StemSpan CC110; StemCell Technologies), at 10^5^ cells/well, in 48-well plates were left untreated or treated for 90 minutes or 18 hours with 5 μM imatinib and/or 5 ng/ml IFNγ, when total cellular RNA and/or total cell lysates were prepared.

### Immunoblotting

Total cell lysates were prepared and analyzed by SDS-polyacrylamide gel electrophoresis and immunoblotting, using mouse anti-STAT1 (clone 42/Stat1; BD Transduction Laboratories; Cat. No.: 610185), rabbit anti-MCL1 antibody (clone D35A5; Cell Signaling Technology (CST); Cat. No.: 5453), rabbit anti-Bcl-XL (clone 54H6; CST; Cat. No.: 2764), rabbit anti-PIM1 (clone C93F2; CST; Cat. No.: 3247), rabbit anti-PIM2 (clone D1D2; CST; Cat. No.: 4730), rabbit anti-PARP9 (Invitrogen; Cat. No.: 40–4400), rabbit anti-PARP14 (Invitrogen; Cat. No.: PA5-78512) and mouse anti-β-actin (clone AC-15; Sigma-Aldrich; Cat. No.: A5441) antibodies.

### IFNγ ELISA

IFNγ concentration was measured in TCMs using the human IFN-gamma DuoSet Elisa (R&D Systems).

### Affymetrix whole transcript array

Total cellular RNA was isolated with Quick RNA MiniPrep Kit (Zymo Research) and quality checked on Agilent Technologies 2200 TapeStation. One hundred nanograms of total RNA were used to prepare cDNA following the GeneChip WT PLUS Reagent Kit labeling protocol. The samples were then hybridized to Human Clariom D arrays and scanned using the Affymetrix GeneChip Scanner 3000 according to standard protocol. Generated CEL files were analyzed using Applied Biosystems Transcriptome Analysis Console (TAC, v4.0) using SST-RMA summarization on gene and exon level.

### Reverse transcription and real-time PCR

Total cellular RNA was isolated with Quick RNA MiniPrep Kit and then reverse transcribed using the SuperScript VILO cDNA Synthesis Kit (Invitrogen). cDNAs were subjected to real-time PCR in a StepOnePlus Real‐Time PCR System, using the Power SYBR Green PCR Master Mix (ThermoFisher Scientific) with primers listed in Table 1. The relative level of mRNA expression was determined with the ΔΔC_T_ method, using EEF1A1 as an endogenous control gene.

**Table 1.** Primers used in real-time PCR.

BCL2L1 long isoform (BCL-XL)	5’-GGTATTGGTGAGTCGGATCG-3’
	5’-TGCTGCATTGTTCCCATAGA-3’
BCL2L3 isoform 1 (MCL-1L)	5’-TCGGTACCTTCGGGAGCA-3’
	5’-TGTCCAGTTTCCGAAGCAT-3’
BCL2L5 (BCL2A1)Isoform 1Isoform 2	5’-AAACGGAGGCTGGGAAAAT-3’
	5’-TGGTCAACAGTATTGCTTCAGG-3’
	5’-CAGGAGAATGGATAAGGCAAA-3’
	5’-CTTCTTGTGGGCCACTGACT-3’
PARP9	5’-CAATGGTCGTGAACAACCTG-3’
	5’-TGCCACAGGTCCAACTGTAA-3’
PARP14	5’-GAGGTTCACTTTCTGCTGCAC-3’
	5’-GCCTTCGGAATTTTGTCACT-3’
PIM1	5’-CAGGCAGAGGGTCTCTTCAG-3’
	5’-TCCATGGATGGTTCTGGATT-3’
PIM2	5’-GGTGGCCATCAAAGTGATTC-3’
	5’-ACCTGCACCCACTTTCCATA-3’
IFI6	5’-AACCGTTTACTCGCTGCTGT-3’
	5’-GCTCCGTCACTAGACCTTGG-3’
EEF1A1	5’-TCCACCACTACTGGCCATCT-3’
	5’-GAGCCCTTTCCCATCTCAG-3’

Primers were purchased from Sigma-Aldrich.

### Analysis of apoptosis using flow cytometry

Cells were stained with PE Active Caspase-3 Apoptosis Kit (BD Biosciences; Cat. No.: 550914) according to the manufacturer`s instructions, followed by the measurement of fluorescence intensities with a NovoCyte 3000 flow cytometer (Acea Biosciences). Data obtained by flow cytometry were analyzed using FlowJo software, version 9.4.11 (Tree star).

### Fluorescence live cell microscopy

Fluorescence live cell microscopy was performed with an IncuCyte S3 Live Cell Analysis System (Essen Bioscience). Nine planes of view were collected per well, using the 20x objective. The obtained data were analyzed with the IncuCyte S3 Cell-by-Cell Analysis Software Module (Essen Bioscience).

### Statistical analysis

Statistical analysis was performed with GraphPad Prism 8.0. Normality of the data was tested using the Kolmogorov–Smirnov test. Paired T-test was used to analyze mean differences between treatments. P < .05 was considered as significant.

## Results

### Activated T cell-conditioned medium strongly counteracts imatinib-induced apoptosis of JURL-MK1 cells, and IFNγ neutralization strongly inhibits this anti-apoptotic effect

**Figure 1.**
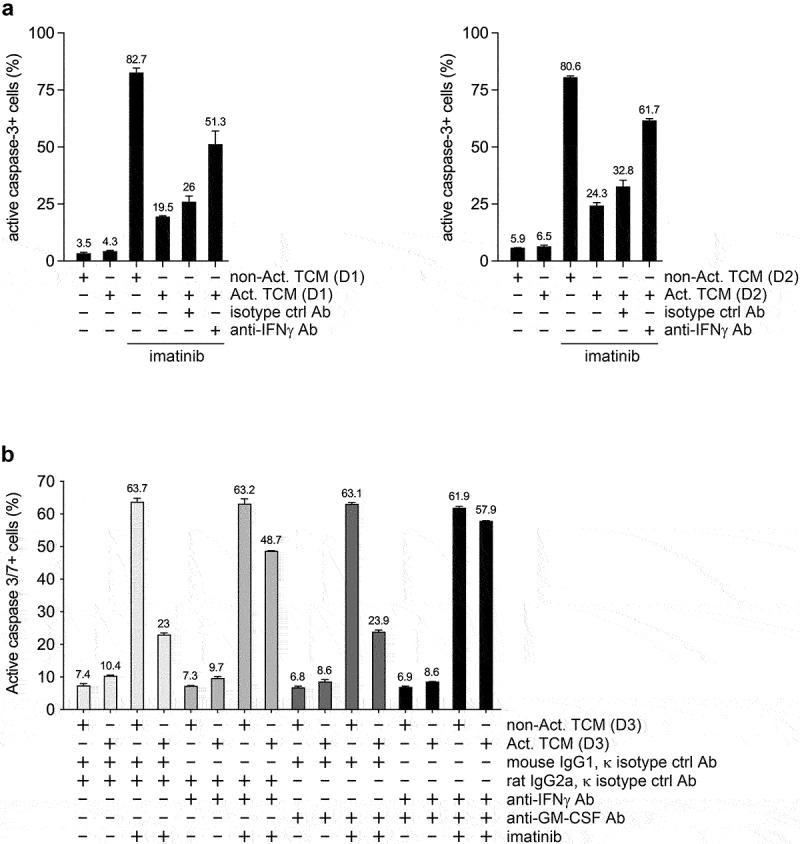
IFNγ neutralization markedly counteracts the anti-apoptotic effect of activated T cell-conditioned medium on imatinib-treated JURL-MK1 cells. (a) Proportion of active caspase-3 positive JURL-MK1 cells left untreated or treated with 1 µM imatinib for 22 hours in a mixture of 20% fresh non-conditioned complete medium and 80% non-activated (non-Act) or activated (Act) TCM (produced in complete medium by T cells enriched from the peripheral blood of two healthy donors; D1, donor No. 1; D2, donor No. 2), in the absence (-) or presence (+) of isotype control (ctrl) or neutralizing anti-human IFNy antibodies (Ab) to a final concentration of 10 μg/ml each, and analyzed by flow cytometry. (b) Proportion of active caspase-3/7 positive JURL-MK1 cells left untreated (-) or treated (+) with 1 µM imatinib for 36 hours in a mixture of 95% fresh non-conditioned complete medium and 5% non-activated (non-Act) or activated (Act) TCM (produced in StemSpan SFEM medium by T cells enriched from the peripheral blood of a third healthy donor; D3, donor No. 3), in the absence (-) or presence (+) of isotype control (ctrl) and/or neutralizing anti-human IFNy and/or anti-human GM-CSF antibodies (Ab) to a final concentration of 20 μg/ml each, and analyzed by fluorescence live cell microscopy. The IFNy concentration in the conditioned mediums produced by the non-activated and activated T cells of donor No. 3 were undetectable and 95,2 ng/ml, respectively. Imatinib-induced apoptosis was faster in the batch (No. 1) of JURL-MK1 shown in panel A than in the batch (No. 2) shown in panel B. Data represent mean with standard deviation derived from three (a) or two (b) technical replicates.

To analyze the role of IFNγ in the effect of activated T cell secretome on imatinib-treated CML cells, we performed IFNγ neutralization experiments on the JURL-MK1 CML cell line using conditioned medium of T cells enriched from PBMCs of healthy blood donors (Figure 1). In the presence of conditioned medium produced by non-activated T cells imatinib induced high rate of apoptosis (as assessed by active caspase-3/7 expression) in JURL-MK1 cells, while the addition of conditioned medium produced by T cells activated by CD3/CD28 beads strongly counteracted the pro-apoptotic effect of imatinib. More importantly, addition of a neutralizing anti-IFNy antibody strongly counteracted the anti-apoptotic effect of activated TCM, although the full anti-apoptotic activity couldn’t be neutralized. However, simultaneous neutralization of IFNγ and GM-CSF nearly completely blocked the anti-apoptotic effect of activated TCM on JURL-MK1 cells (Figure 1b), demonstrating that the anti-apoptotic activity of the activated T cell secretome on JURL-MK1 cells is mediated mainly through IFNγ, and to a lesser extent via GM-CSF.

### IFNγ rapidly exerts its anti-apoptotic effect on imatinib treated CML cells

**Figure 2.**
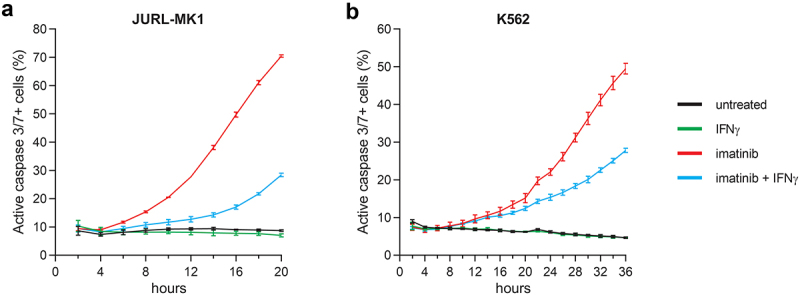
IFNγ exerts a rapid anti-apoptotic effect on imatinib-treated CML cells. Proportion of active caspase-3/7 positive JURL-MK1 (batch No. 1) (a) and K562 (b) cells left untreated or treated for the hours indicated with 1 μM imatinib and/or 5 ng/ml IFNγ, analyzed by fluorescence live cell microscopy. Black line, untreated; green line, IFNγ; red line, imatinib; blue line, imatinib and IFNγ. Data represent mean with standard deviation derived from three technical replicates. Experiments were performed at least twice with similar results.

To characterize the kinetics of the anti-apoptotic effect of IFNγ on CML cells, the proportion of active caspase-3/7 positive cells was analyzed in JURL-MK1 and K562 cells left untreated or treated with imatinib and/or IFNγ with fluorescence live cell microscopy (Figure 2). Imatinib treatment induced rapid apoptosis of JURL-MK1 cells, while the pro-apoptotic response was slower in K562 cells. The anti-apoptotic effect of IFNγ was already detectable at the start of imatinib-induced apoptosis in JURL-MK1 cells, while it was delayed with a few hours in K562 cells.

### Reduction of STAT1 protein levels markedly counteracts the anti-apoptotic effect of IFNγ on imatinib-treated CML cells

**Figure 3.**
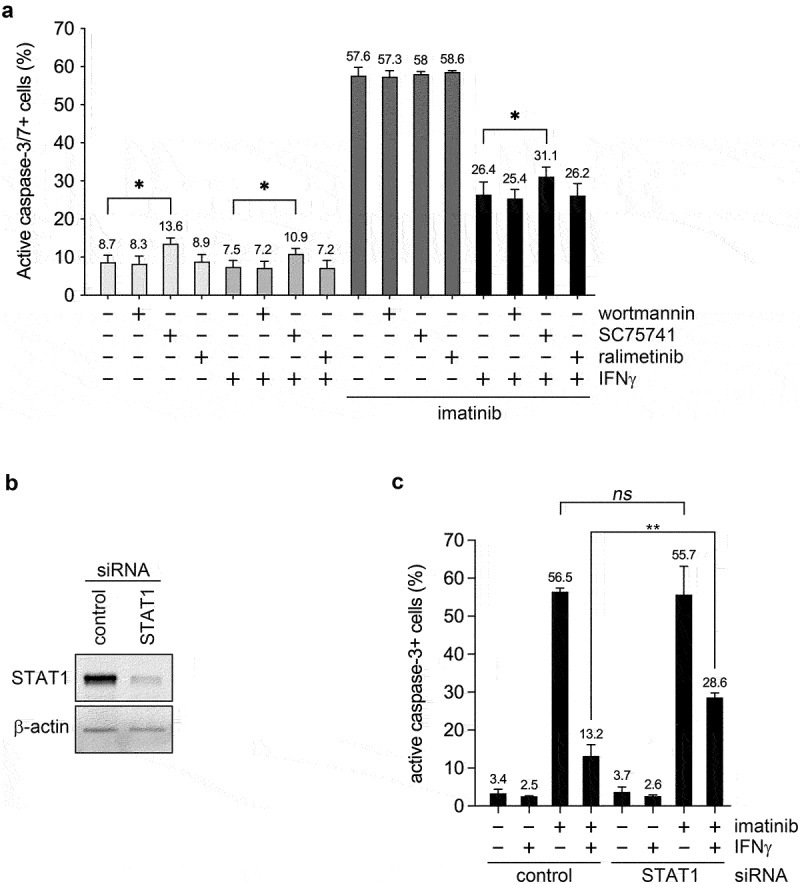
Reducing STAT1 protein level markedly inhibits the anti-apoptotic effect of IFNγ on imatinib-treated JURL-MK1 cells. (a) Proportion of active caspase-3/7 positive JURL-MK1 (batch No. 1) cells left untreated (-) or treated (+) for 18 hours with 1 μM imatinib and/or 5 ng/ml IFNγ in the absence (-) or presence (+) of 100 nM wortmannin or 10 μM SC75741 or 500 nM ralimetinib, analyzed by fluorescence live cell microscopy. (b) Immunoblot analysis of STAT1 and β-actin protein expression in total cell extracts of JURL-MK1 (batch No. 1) cells transfected with a negative control or a STAT1-specific siRNA. (c) Proportion of active caspase-3 positive JURL-MK1 (batch No. 1) cells transfected with a negative control or a STAT1-specific siRNA, and then left untreated (-) or treated (+) for 18 hours with 1 μM imatinib and/or 5 ng/ml IFNγ. Data represent mean with standard deviation derived from three (a) or four (c) independent experiments. Representative blots are shown. ns, not significant; *P < .05; **P < .01.

IFNγ is known to activate several alternate pathways in addition to the classical JAK/STAT1 signaling.^17^ Since inhibition of phosphatidylinositol 3´-kinase (PI3K), extracellular signal-regulated kinases (ERK1/2), p38 mitogen-activated protein kinase, c-Jun N-terminal kinases (JNK1/2/3) and NF-κB (with 100 nM wortmannin, 2 μM SCH772984, 500 nM ralimetinib, 2.5 μM JNK-IN-8 and 10 μM SC75741, respectively) only minimally affected or did not prevent at all the anti-apoptotic effect of IFNγ on imatinib-treated JURL-MK1 cells (Figure 3a and data not shown), we focused our analysis on the role of the classical STAT1 signaling pathway. Therefore, we measured the extent of apoptosis in imatinib- and/or IFNγ-treated JURL-MK1 cells previously transfected with either a negative control or a STAT1-specific siRNA. Since transient reduction of STAT1 protein levels with a siRNA markedly counteracted the anti-apoptotic effect of IFNγ on imatinib-treated JURL-MK1 cells (Figure 3b), we conclude that the classical STAT1 pathway plays an important role in the anti-apoptotic effect of IFNγ on CML cells.

### Activated T cell-conditioned medium increases proliferation and counteracts imatinib-induced apoptosis of primary human CD34+ CML stem/progenitor cells

**Figure 4.**
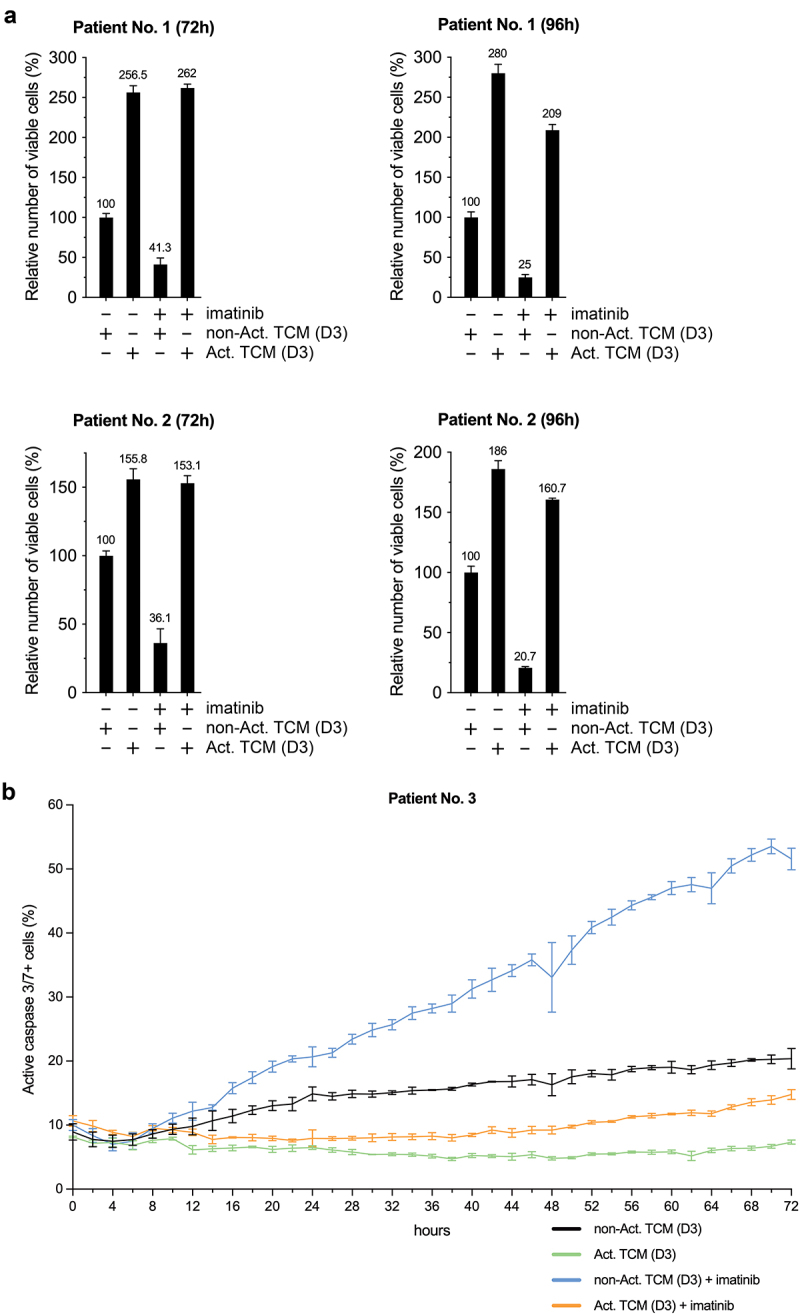
Activated T cell-conditioned medium increases proliferation and counteracts imatinib-induced apoptosis of primary human CD34+ CML stem/progenitor cells. Relative numbers of viable (a), and proportion of active caspase-3/7 positive (b) primary human CD34+ CML stem/progenitor cells (obtained from the peripheral blood of Patients Nos. 1, 2 (a) and 3 (b)), left untreated or treated for the hours indicated with 5 μM imatinib in a mixture of 80% fresh non-conditioned StemSpan SFEM medium and 20% non-activated (non-Act) or activated (Act) TCM (the same TCMs (D3) used in the experiment shown in Figure 1b), and analyzed by cell counting (a) or fluorescence live cell microscopy (b). Black line, non-activated TCM; green line, activated TCM; blue line, non-activated TCM and imatinib; orange line, activated TCM and imatinib. Data represent mean with standard deviation derived from three technical replicates.

Next, we analyzed the effects of the activated T cell secretome (with the same TCMs used in the experiment shown in Figure 1b) on the proliferation and apoptosis of untreated and imatinib-treated primary human CD34+ CML stem/progenitor cells. Similar to the results obtained on JURL-MK1 cells, activated TCM markedly increased the number of viable (Figure 4a) and decreased the rate of apoptotic (active caspase-3/7 positive) (Figure 4b) CML stem/progenitor cells, both in the absence or presence of imatinib.

### IFNγ neutralization strongly counteracts the anti-apoptotic effect of activated T cell secretome on primary human CD34+ CML stem/progenitor cells during the first 36 hours, while at later time-points, simultaneous IFNγ and GM-CSF neutralization partially blocks the anti-apoptotic effect

**Figure 5.**
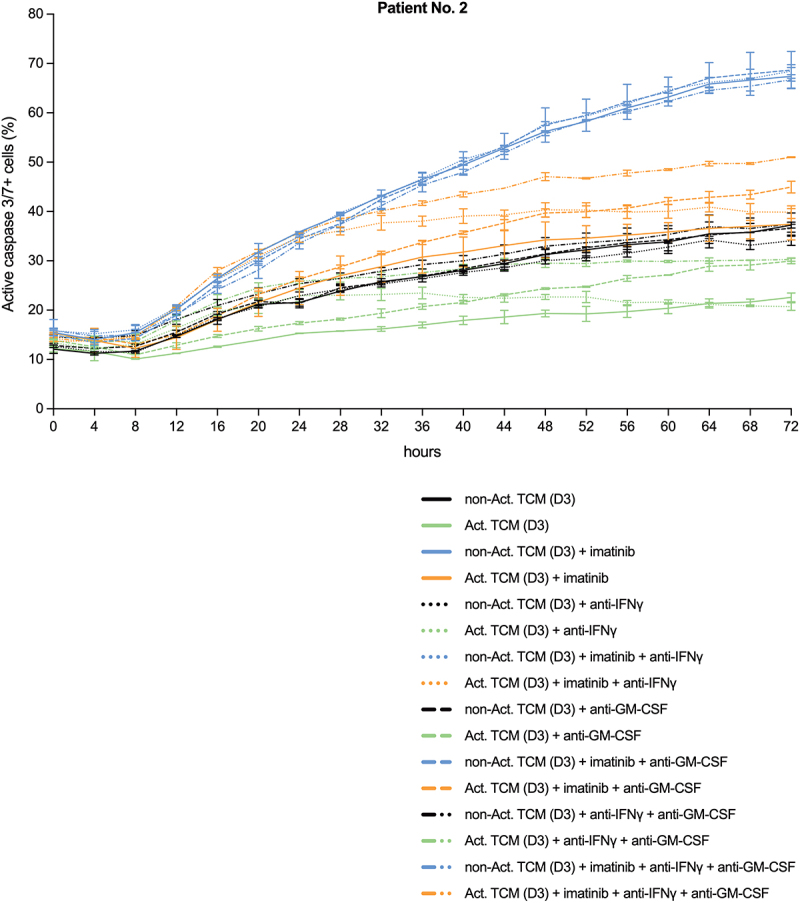
The anti-apoptotic effect of activated T cell secretome on primary human CD34+ CML stem/progenitor cells is strongly counteracted by IFNγ neutralization during the first 36 hours, while at later time-points simultaneous IFNγ and GM-CSF neutralization partially blocks the anti-apoptotic effect. Proportion of active caspase-3/7 positive primary human CD34+ CML stem/progenitor cells (obtained from the peripheral blood of Patient No. 2) left untreated or treated for the indicated hours with 5 µM imatinib in a mixture of 95% fresh non-conditioned StemSpan SFEM medium and 5% non-activated (non-Act) or activated (Act) TCM (the same TCMs (D3) used in the experiments shown in Figure 1b and Figure 4), in the presence of only the two isotype controls (solid lines), or neutralizing anti-human IFNy and rat IgG2a isotype control (dotted lines), or neutralizing anti-human GM-CSF and mouse IgG1 isotype control (dashed lines), or the combination (dash-dotted lines) of the two neutralizing antibodies (Ab) to a final concentration of 20 μg/ml each, and analyzed by fluorescence live cell microscopy. Black line, non-activated TCM; green line, activated TCM; blue line, non-activated TCM and imatinib; orange line, activated TCM and imatinib. Data represent mean with standard deviation derived from two technical replicates.

Next, the neutralization experiment performed on JURL-MK1 cells (Figure 1b) was repeated (with the same TCMs) on primary human CD34+ CML stem/progenitor cells (Figure 5). Interestingly, IFNγ proved to be the key anti-apoptotic cytokine only during the first 36 hours of the treatment, while at later time-points, simultaneous IFNγ and GM-CSF neutralization only partially counteracted the anti-apoptotic effect, suggesting that other unknown factor(s) might also be important anti-apoptotic component(s) of the activated T cell secretome.

### The anti-apoptotic effect of IFNγ on primary human CD34+ CML stem/progenitor cells is general and only partially depends on MCL1 upregulation

**Figure 6.**
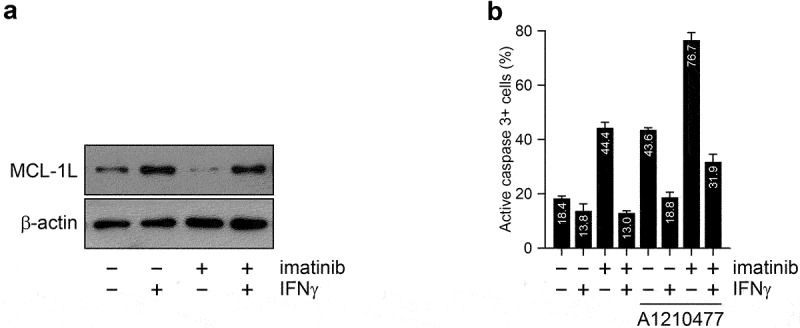
The MCL1 inhibitor A1210477 only partially blocks the anti-apoptotic effect of IFNγ on imatinib-treated primary human CD34+ CML stem/progenitor cells (a) Immunoblot analysis of MCL-1L and β-actin protein expression in total cell extracts of primary human CD34+ CML stem/progenitor cells (obtained from the peripheral blood of Patient No. 4) left untreated (-) or treated (+) for 18 hours with 5 μM imatinib and/or 5 ng/ml IFNγ. (b) Proportion of activated caspase-3 positive primary human CD34+ CML stem/progenitor cells (obtained from the peripheral blood of Patient No. 4) left untreated (-) or treated (+) for 24 hours with 5 μM imatinib and/or 5 ng/ml IFNγ in the absence or presence of 10 μM A1210477. Data represent mean with standard deviation derived from four technical replicates.

We have previously shown an essential role for MCL-1L upregulation in the anti-apoptotic effect of IFNγ on imatinib treated JURL-MK1 cells.^13^ In line with this observation, IFNγ strongly upregulated MCL-1L expression and completely inhibited the pro-apoptotic effect of imatinib in primary human CD34+ CML stem/progenitor cells (Figure 6). However, the presence of A-1210477 only minimally inhibited the anti-apoptotic effect of IFNγ on imatinib treated CML stem/progenitor cells, and even the apoptosis induced by A-1210477 monotherapy was completely counteracted by IFNγ in these cells (Figure 6b). These results suggest that IFNγ has a general anti-apoptotic effect on CML stem/progenitor cells, and that it might exert this effect through additional mechanisms, besides MCL-1L upregulation.

### IFNγ upregulates the expression of several key anti-apoptotic proteins in imatinib-treated primary human CD34+ stem/progenitor cells

**Figure 7.**
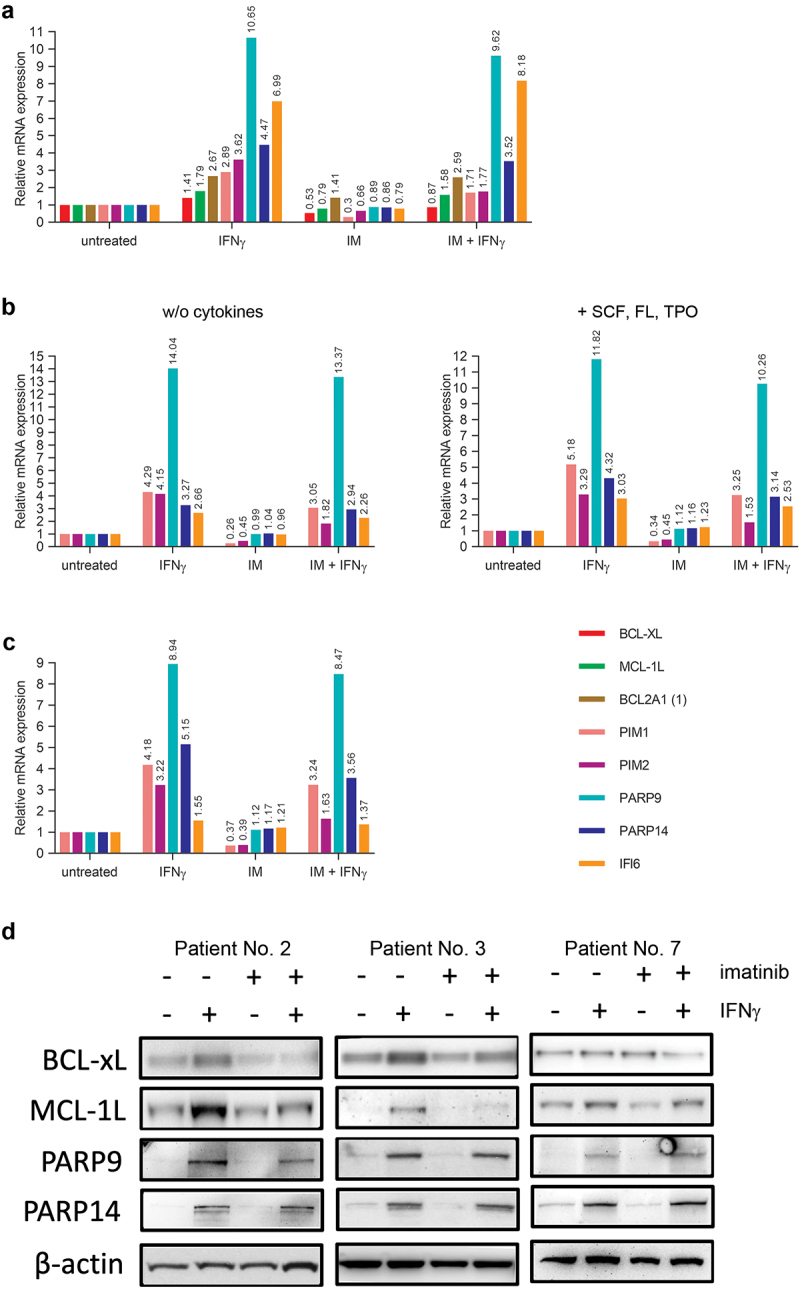
IFNγ upregulates the expression of several key anti-apoptotic genes in imatinib-treated primary human CD34+ CML stem/progenitor cells. (a) Relative mRNA levels of selected key anti-apoptotic genes normalized to EEF1A1, quantified by real-time RT-PCR in primary human CD34+ CML stem/progenitor cells (obtained from the peripheral blood of Patient No. 4) left untreated or treated for 18 hours with 5 μM imatinib (IM) and/or 5 ng/ml IFNγ. (b) Relative mRNA levels of selected key anti-apoptotic genes normalized to EEF1A1, quantified by real-time RT-PCR in primary human CD34+ CML stem/progenitor cells (obtained from the peripheral blood of Patient No. 5) left untreated or treated for 90 minutes with 5 μM imatinib (IM) and/or 5 ng/ml IFNγ in the absence (w/o cytokines) or presence of SCF, FL, and TPO. (c) Relative mRNA levels of selected key anti-apoptotic genes normalized to EEF1A1, quantified by real-time RT-PCR in primary human CD34+ CML stem/progenitor cells (obtained from the peripheral blood of Patient No. 6) left untreated or treated for 90 minutes with 5 μM imatinib (IM) and/or 5 ng/ml IFNγ in the absence of cytokines. (d) Immunoblot analysis of BCL-XL, MCL-1L, PARP9, PARP14 and β-actin protein expression in total cell extracts of CD34+ stem/progenitor cells (obtained from the peripheral blood of Patients Nos. 2, 3 and 7) left untreated (-) or treated (+) for 18 hours with 5 μM imatinib and/or 5 ng/ml IFNγ. PIM1 and PIM2 proteins were expressed in extremely low amounts, or were not detectable in any of the lysates (not shown).

To reveal the molecular details of the anti-apoptotic effect of IFNγ on CML stem/progenitor cells, we analyzed the effect of IFNγ treatment on the transcriptome of imatinib-treated primary human CD34+ CML stem/progenitor cells with an Affymetrix whole transcript array (data not shown), followed by validation with real time reverse transcription PCR. We found, that 18 hours of IFNγ treatment both in the absence and presence of imatinib markedly upregulated the mRNA expression of several key anti-apoptotic genes beside MCL-1L (Figure 7a), including BCL-XL and BCL2A1 (additional anti-apoptotic members of the BCL2 gene family),^18^ the proviral integration site for Moloney Murine Leukemia virus (PIM) kinases PIM1 and PIM2,^19^ PARP9,^20^ PARP14^21^ and IFNα inducible protein 6 (IFI6, G1P3).^22,23^ Since the anti-apoptotic effect of IFNγ proved to be rapid in CML cells, we also analyzed the expression of these genes in CD34+ CML stem/progenitor cells (obtained from two additional CML patients) left untreated or treated for 90 minutes with IFNγ and/or imatinib (Figure 7b and 7c). This short IFNγ treatment only minimally affected MCL-1L, BCL-XL and BCL2A1 mRNA levels (data not shown), but strongly upregulated PIM1, PIM2, PARP9, and PARP14 mRNA expression both in the absence or presence of imatinib in both of the patient samples, while IFI6 showed similar upregulation only in one of the patient samples.

Analysis of MCL-1L, BCL-XL, PIM1, PIM2, PARP9, and PARP14 protein expression with western blot showed that 18 hours of IFNγ treatment both in the absence or presence of imatinib consistently upregulated MCL-1L, PARP9, and PARP14 protein expression in primary human CD34+ CML stem/progenitor cells obtained from three additional patients. On the other hand, PIM1 and PIM2 proteins could not be detected or were only minimally expressed, and BCL-XL protein expression did not show any consistent response upon IFNγ treatment in the analyzed samples (Figure 7d).

Since the rapidly upregulated PARP9 and PARP14 genes encode key survival proteins with strong anti-apoptotic effects in hematopoietic cells,^20,21^ their prompt simultaneous upregulation followed by the delayed induction of MCL-1L expression might explain the marked anti-apoptotic effect of IFNγ on imatinib treated CML stem/progenitor cells.

## Discussion

Several lines of evidence suggest a key role for IFNγ in the mechanisms behind resistance to TKI treatment in CML patients, a remaining problem of major clinical significance. While IFNγ seems capable of acting as an anti-tumorigenic factor in CML patients (indirectly, through the activation of a multitude of cellular components of the immune system),^24^ several publications show that it can also exert direct anti-apoptotic, pro-proliferative and pro-clonogenic effects on CML cells, both in the absence and presence of TKIs.^12,13,25^

Held *et al*.^12^ demonstrated that soluble factors produced by activated T or NK cells significantly inhibited the imatinib- or nilotinib-induced apoptosis of CML cell lines and PBMCs of untreated CML patients in chronic phase. In contrast, non-activated T or NK cells did not exert this effect. Since among the several cytokines tested, only IFNγ counteracted the pro-apoptotic effect of TKIs on CML cells, the authors suggested that IFNγ might be the key soluble anti-apoptotic factor produced by activated T and NK cells, although this assumption has not been proven by neutralization experiments. Furthermore, Schurch *et al*.^25^ showed that transfer of activated leukemia-specific effector cytotoxic T cells into a mice model of CML induced the proliferation of leukemic stem cells through IFNγ secretion, while IFNγ treatment *in vitro* increased the proliferation and colony formation of primary human CD34+ CML stem/progenitor cells. We now show that activated TCM exerts strong pro-proliferative and anti-apoptotic effects on CML cells (including primary human CD34+ CML stem/progenitor cells). Experiments with neutralizing antibodies showed that at early time-points IFNγ is a major, while GM-CSF is a minor anti-apoptotic component of the activated T cell secretome. However, at later time-points, simultaneous neutralization of these two cytokines only partially blocked the anti-apoptotic effect of the activated TCM on CML stem/progenitor cells, suggesting that additional soluble factors may also contribute to the noted effect.

Previous reports^12,13^ demonstrated that exogenously added IFNγ exert strong anti-apoptotic effect on CML cell lines and PBMCs of CML patients, but did not analyze its anti-apoptotic effect on CML stem/progenitor cells, the cell population responsible for residual disease.^1,2^ Our results now revealed that exogenously added IFNγ markedly counteracts imatinib-induced apoptosis of CML stem/progenitor cells.

Using chemical inhibitors or siRNA knockdown we could demonstrate that the major alternative IFNγ pathways, i.e. PI3K, p38, ERK1/2, JNK1/2/3 and NF-κB, are not essential, while STAT1 signaling plays a significant role in the anti-apoptotic effect of IFNγ on CML cells. This result was unexpected, as STAT1 signaling is generally considered to be pro-apoptotic and anti-proliferative. On the other hand, STAT1 signaling has also been shown to exert anti-apoptotic and pro-proliferative effects in certain cancer types.^26,27^ The molecular mechanism, by which STAT1 signaling exert divergent effects in different cancer and/or cell types is however not well known and needs to be further elucidated.

We have previously shown that IFNγ upregulates the expression of several genes with potential anti-apoptotic function, including BCL6 and MCL-1L, in JURL-MK1 cells. We have also shown that *ex vivo* IFNγ treatment enhanced in a BCL6-dependent manner the cluster formation of imatinib-treated primary human CD34+ CML stem/progenitor cells. On the other hand, BCL6 knockdown did not inhibit, while the presence of 10 μM A-1210477 completely counteracted the anti-apoptotic effect of IFNγ on imatinib-treated JURL-MK1 cells.^13^ We now show that although MCL-1L is upregulated by IFNγ in CML stem/progenitor cells, inhibition of MCL1 activity with 10 μM A-1210477 only partially counteract the anti-apoptotic effect of IFNγ in these cells. This result suggests that other mechanisms may also contribute to the observed anti-apoptotic effect of IFNγ. Although genome-wide analysis of mRNA expression revealed that IFNγ strongly upregulated several key anti-apoptotic genes in imatinib-treated CML stem/progenitor cells, including MCL-1L, BCL-XL, BCL2A1, PIM1, PIM2, PARP9, PARP14, and IFI6, western blot analysis could confirm the IFNγ-induced consistent upregulation of only MCL-1L, PARP9, and PARP14. MCL1 is a key anti-apoptotic member of the BCL2 gene family, that is frequently overexpressed in several hematopoietic cancer types, including CML.^18,28,29^ PARP9 (B-aggressive lymphoma-1; BAL1) plays an essential role in the survival of a subclass of high-risk diffuse large B cell lymphomas associated with a prominent inflammatory infiltrate.^20^ The macro-PARP subfamily member PARP14 is a binding partner of STAT6 and plays a central role in the anti-apoptotic effect of IL-4 on B cells.^21^ These well-characterized effects on various hematopoietic cell types suggest that the concomitant upregulation of these genes might explain the strong anti-apoptotic effect of IFNγ on CML stem/progenitor cells. Identification of the key actors in this complex interplay balancing pro- and anti-apoptotic events related to TKI treatment will be of paramount interest in the quest to successfully eradicate residual leukemic cells and thereby achieving cure of CML.

## Data Availability

Additional data that support the findings of this study are available from the corresponding author, DS, upon reasonable request.
